# A cross-sectional study of viral load suppression among youth living with HIV in Namibia

**DOI:** 10.4102/sajhivmed.v27i1.1742

**Published:** 2026-01-30

**Authors:** Jacques W.N. Kamangu, Sheillah H. Mboweni

**Affiliations:** 1 Department of Health Studies, College of Human Sciences, University of South Africa, Pretoria, South Africa

**Keywords:** adherence, older adolescents, antiretroviral therapy, HIV, viral load suppression, younger adults

## Abstract

**Background:**

Meeting the Joint United Nations Programme on HIV/AIDS targets and ending AIDS by 2030 requires global efforts, with a particular focus on older adolescents living with HIV (OALHIV) and younger adults living with HIV (YALHIV). These population groups are often associated with unsuppressed viral load compared to adults.

**Objectives:**

This article assessed the extent of viral load suppression (VLS) and associated factors among this group in seven high-burden districts of Namibia.

**Method:**

A cross-sectional survey was conducted to analyse data for 600 OALHIV and YALHIV, aged 15–24 years, who were already on antiretroviral treatment prior to 2020. The study employed a stratified cluster sampling across seven districts. Data were extracted from an electronic database and analysed using Statistical Package for Social Sciences (SPSS) software. However, limitations within the database restricted the availability of certain variables.

**Results:**

Overall, the VLS (< 1000 copies/mL) was 84.8%, with female patients showing a significantly higher VLS 88.3% compared to male patients, with 78.5% (*P* < 0.01; odds ratio [OR] = 2.08). Although OALHIV had higher suppression (84.9%) than YALHIV (74.6%), age was not significantly associated with VLS (*P* = 0.9). Dolutegravir-based regimens had a suppression of 93.3% (*P* < 0.01; OR = 9.1), and those with a fixed home address had an 88.2% suppression (*P* = 0.014; OR = 1.76).

**Conclusion:**

The VLS of 84.8% remains below the Joint United Nations Programme on HIV/AIDS target of 95%. There is a need for improvements in antiretroviral treatment programmes, particularly in scaling up dolutegravir-based regimens, enhancing adherence and peer support to end AIDS by 2030.

**What this study adds:** This study offers insights into viral load suppression among older adolescents and YALHIV in Namibia. These populations remain vulnerable to poor treatment outcomes.

## Introduction

Viral load suppression (VLS) is essential for effective HIV management and reducing transmission.^1^ Despite global progress in antiretroviral therapy (ART) access, older adolescents living with HIV (OALHIV) and younger adults living with HIV (YALHIV) still face challenges in achieving optimal VLS.^1,2^ These population groups, encompassing individuals aged 15 years to 24 years, face social, psychological, and economic changes that can affect their adherence to HIV treatment.^3^

There are various definitions of VLS in the context of HIV care and treatment. Some literature defines VLS as the reduction of viral load (VL) to undetectable levels, typically below 40 copies/mL,^1^ an approach also adopted by Namibia, while the WHO defines VLS as VL of < 1000 copies/mL.^4^ Other sources set the threshold at 400 copies/mL, which is generally considered indicative of low level of viraemia (LLV),^5,6^ as it falls between 40 and < 1000 copies/mL.^7^ However, studies have shown that OALHIV and YALHIV often struggle to achieve and maintain viral suppression, impacting their long-term health and HIV epidemic control.^8^

Approximately 37.7 million individuals worldwide are living with HIV, with around 1.75 million being adolescents aged 10 to 19 years.^7^ Nearly 90% of these adolescents are located in sub-Saharan Africa.^9^ Moreover, HIV poses a greater concern within the population of children and adolescents, comprising just 5% of people living with HIV (PLWH) but accounting for 15% of deaths related to AIDS.^10^ Older adolescents living with HIV and YALHIV face unique socio-economic, psychological, and healthcare challenges that affect treatment adherence and VLS.^2^

As of 2023, Namibia had an estimated 230 000 PLWH, the majority of whom were adults aged 15 years and older. Among them, 7700 were children under 15, and 19 970 were youth aged 15–24. The national HIV prevalence among individuals aged 15–49 was 9.0%, with a notable gender disparity.^11^ The country has made strong progress in its HIV response, with new infections dropping by 78% since 2000. In 2024, 4500 new cases were reported and 66% among adult women, and fewer than 500 in children. The country exceeded the Joint United Nations Programme on HIV/AIDS (UNAIDS) 95–95–95 targets, reaching 96–98–98% among adults. However, cascade outcomes for children, adolescents, and young adults remain lower and highlighting the need for targeted interventions.^11^

Despite the introduction of ART access since 2002, achieving optimal VLS among OALHIV and YALHIV remains challenging. Understanding VLS in this group is key to guiding targeted interventions and improving outcomes.^12,13^ Additionally, despite support from partners such as the United States President’s Emergency Plan for AIDS Relief and the Centers for Disease Control to strengthen the national HIV response, most interventions have focused on adults in high-burden regions, with limited attention to the unique needs of OALHIV and YALHIV. In Namibia, it is estimated that 19 670 individuals fall within this population group, comprising 7015 (35.6%) OALHIV and 12 655 (64.3%) YALHIV. Their VLS stands at 84%, falling below national targets and underscoring the need for age-specific strategies.^14,15^

This study assesses VLS among OALHIV and YALHIV in Namibia to inform targeted, evidence-based interventions that enhance treatment outcomes and reduce HIV transmission.

## Research methods and design

### Research design

A quantitative descriptive cross-sectional study was employed to assess the extent of VLS among OALHIV and YALHIV. These studies are frequently compared to a ‘snapshot’ of a population at a particular moment, as they involve the simultaneous determination of both exposure and outcome for each subject.^16^

### Research setting

The research took place in six Namibian regions: Khomas, Ohangwena, Oshana, Omusati, Zambezi, and Oshikoto, with particular attention to seven districts with high burdens of HIV. These districts constituted 70% of the target population and housed the majority of PLWH with a concentration in the northern and peri-urban areas characterised by lower socio-economic status (Engela, Outapi, Oshikuku, Oshakati, Onandjokwe & Katima districts), as opposed to the fully urban area in the central part of the country (Windhoek).^17^

### Population and sampling

In this study, routine records of OALHIV and YALHIV who were active on ART and enrolled into care before January 2020 were included. This criterion ensured sufficient treatment duration prior to data collection in May 2022 and allowed for adequate documentation of VL monitoring and follow-up interventions. Only records with at least one valid VL result recorded in the electronic patient monitoring system (ePMS) were considered. Data were drawn from 90 randomly selected health facilities out of 113 across seven high-burden districts, exceeding the minimum required sample size of 88 facilities. The ePMS remains the primary database for all PLWH on treatment in the public sector in Namibia. To ensure data validity, records extracted at the central level were cross-checked against data from some selected health facilities for the same reporting period.

A probability-based stratified cluster sampling method combining cluster, stratified, and random sampling techniques was used to select records of OALHIV and YALHIV for this study. Six regions were grouped to include seven high-burden districts, within which health facilities were stratified and randomly selected. From a total population of 19 670 OALHIV and YALHIV records across 90 facilities, a random sample of 600 records was drawn using the Raosoft sample size calculator (Raosoft, Inc., Seattle, Washington, United States), based on a 5% margin of error and a 95% confidence level.^18^

### Data collection method

To collect the necessary information for the study, a checklist was utilised, which was developed in collaboration with statisticians who had expertise in working with the ePMS. Additionally, the team lead of the research monitoring and evaluation division, responsible for hosting the ePMS, guided the checklist development process.

### Data analysis

Descriptive and inferential analyses were conducted using Statistical Package for Social Sciences (SPSS) version 28 (IBM Corp., Armonk, New York, United States), to determine the prevalence of VLS among OALHIV and YALHIV based on their most recent VL results documented in the ePMS. This statistical analysis served as the initial stage of analysis, aiding researchers in summarising data and identifying patterns. It explained the attributes of a population, encompassing measures like mean, mode, median, percentage, frequency, and range for a given variable.^19^ The data were entered in a Microsoft Excel sheet, and the cleaning and coding were done by the researchers to structure the data. Thereafter, the structure data was exported into SPSS. This approach is particularly valuable in contemporary research, where large data sets may make it challenging to discern fundamental relationships within the collected information.^20^

Crude and adjusted regression analyses were used to identify various factors associated with VLS. Because of regimen documentation issues, the analysis was not done based on the regimen lines (first, second, and third), to avoid misclassification biases. Independent variables such as age and VL were still analysed using descriptive and inferential statistics. To align with international standards, the authors employed the VLS cut-off of < 1000 copies/mL per, as per the WHO’s definition.^1,4^

### Ethical considerations

The University of South Africa Research Ethics committee provided the approval for this study protocol (NHREC registration number: Rec-240816-052; CREC reference number: 12786918_CREC_CHS_2021). Permission to conduct the study at the seven high-burden districts was granted by the Ministry of Health and Social Service research committee (reference number 17/3/3/JK). Informed consent was not part of the requirement from OALHIV and YALHIV, as there was no direct contact with them. Confidentiality was maintained during the data collection and analysis and only the researchers had accessed the databases. The anonymity was ensured by using the ART unique number rather than their names and coding was used during data analysis.

## Results

Records were gathered from the ePMS for 600 active OALHIV and YALHIV who were receiving ART care in seven high-burden districts. Among this group, 281 individuals (46.8%) initiated their ART treatment before 2010, while 231 individuals (38.5%) commenced treatment between 2010 and 2018. Only 88 individuals (14.7%) who initiated treatment in 2019 or later were started on dolutegravir, the recommended optimal regimen for adolescents living with HIV experiencing adherence challenges.

### Participants’ characteristics

There was a significant gender disparity, with 63.3% (*n* = 380/600) female patients (*P* < 0.01), reflecting the broader gender distribution among PLWH in Namibia. The median age was 20.0 years, with no significant difference by gender (*P* = 0.9). The majority of patients (86%, *n* = 516/600) did not own cell phones. Additionally, 52.5% (*n* = 315/600) had a fixed home address (*P* = 0.014). Most patients (71.8%, *n* = 431/600) were on a dolutegravir regimen which was introduced in December 2019 in Namibia, as shown in Table 1.

**TABLE 1:** Determinants of viral load suppression for older adolescents and younger adults on antiretroviral therapy in the seven high-burden districts in Namibia, May 2023.

Variables	*n*	% VLS (Namibian definition)	% LLV	% VLS (WHO definition)	% HVL	*P*
**Participants**	600	71.8	13.0	84.8	15.1	-
**Districts**	-	-	-	-	-	0.540
Windhoek	107	71.0	14.0	85.0	15.0	-
Engela	96	64.0	17.0	81.0	19.0	-
Outapi	70	77.0	8.0	85.0	15.0	-
Oshikuku	94	75.0	16.0	91.0	9.0	-
Oshakati	100	74.0	9.0	83.0	17.0	-
Onandjokwe	35	68.0	14.0	82.0	18.0	-
Katima	98	71.0	11.0	82.0	18.0	-
**Gender**	-	-	-	-	-	< 0.010
Male	220	63.1	15.4	78.5	21.3	-
Female	380	76.8	11.5	88.3	11.5	-
**Age (years)**	-	-	-	-	-	0.900
15–19	254	70.4	14.5	84.9	14.5	-
20–24	346	72.8	11.8	74.6	15.3	-
**On dolutegravir regimen**	-	-	-	-	-	< 0.010
Yes	431	83.7	9.9	93.6	6.2	-
No	169	41.4	20.7	62.1	37.8	-
**Type of treatment**	-	-	-	-	-	0.270
On initial treatment	85	68.2	14.1	82.3	17.6	-
Switch	77	50.6	14.2	64.8	35.0	-
Substitution	438	75.1	15.0	90.1	9.8	-
**Fixed home address**	-	-	-	-	-	0.014
Yes	315	75.2	13.0	88.2	11.7	-
No	285	68.0	12.9	80.9	18.9	-
**Year of ART start**	-	-	-	-	-	0.160
Before 2010	281	68.6	13.8	85.2	17.4	-
2010–2018	231	70.5	14.7	85.2	14.7	-
2019 up to May 2022	88	85.2	5.6	90.8	9.0	-
**Documented cell phone possession**	-	-	-	-	-	0.370
Yes	84	75.0	13.0	88.0	11.9	-
No	516	71.3	12.9	84.4	15.6	-

ART, antiretroviral therapy; VLS, viral load suppression; LLV, low-level viraemia; HVL, high viral load.

### Viral load outcome and associated factors

The VL results were categorised into four groups: virologic suppression with VL levels below 40 copies/mL per Namibian definition, LLV with VL between 40 copies/mL and 1000 copies/mL, virologic suppression with VL levels below 1000 copies/mL per WHO definition and high VL (HVL) with VL from 1000 copies/mL and above. The outcome demonstrated that 431 records (71.8%) of OALHIV and YALHIV were virally suppressed, while 78 records (13%) fell into the category of LLV, and 91 records (15.2%) exhibited HVL. When combining VLS with LLV (VL < 1000 copies/mL), the total number of OALHIV and YALHIV records meeting the WHO definition of virologic suppression was 509 (84.8%).

**FIGURE 1:**
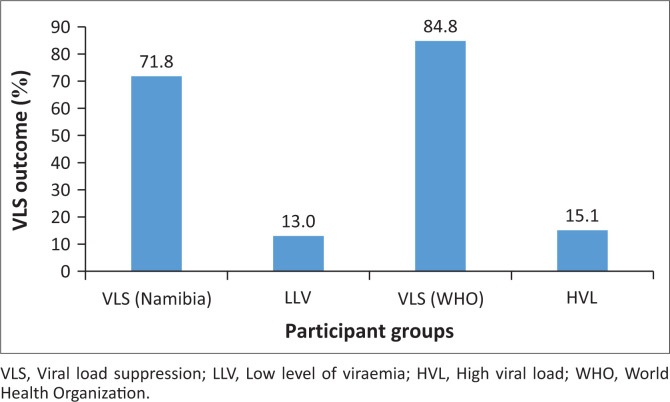
Participants’ overall viral load suppression.

VLS was 84.8% (95% confidence interval: 81.2% – 87.3%) with no statistical difference across the seven high-burden districts, with a *P*-value of 0.54 (Figure 1).

**FIGURE 2:**
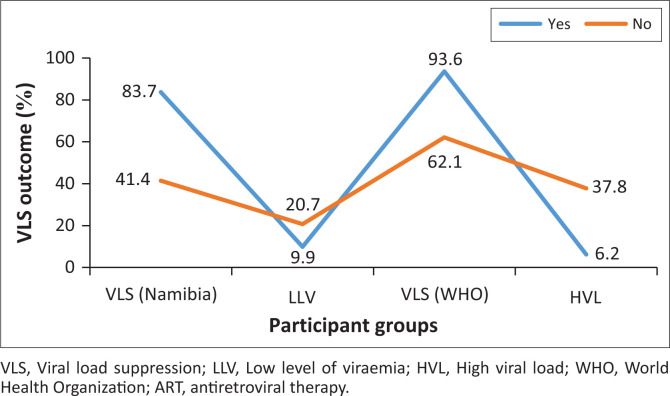
Viral load suppression outcome of dolutegravir-based regimens with other ART regimens.

The initiation of the dolutegravir-based regimen began in mid-2019, with transitioning or regimen changes guided by patients’ VL values. While the transitioning was largely completed early in January 2020, with an ongoing regimen change in cases of failure to treatment, records showed that OALHIV and YALHIV on dolutegravir-based regimens achieved significantly better VLS compared to those on non-dolutegravir-based regimens (*P* = 0.01; odds ratio [OR] = 9.1), as seen in Figure 2 and Table 1.

**FIGURE 3:**
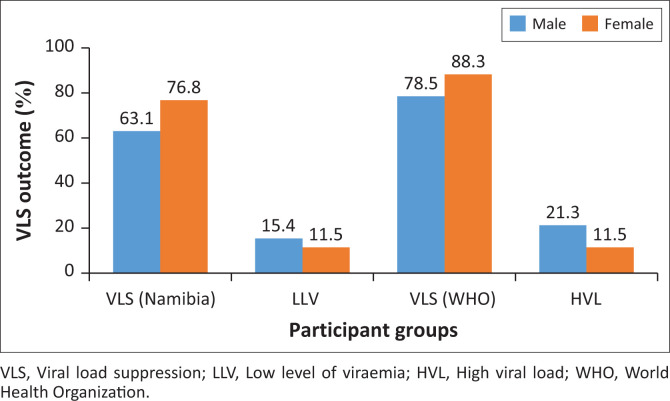
Viral load suppression outcome and gender.

**FIGURE 4:**
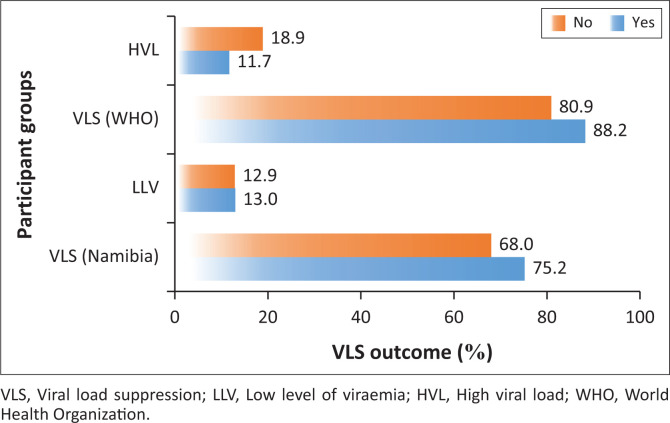
Viral load suppression outcome and fixed home address.

In addition to the dolutegravir-based regimen, other variables that exhibited statistically significant associations with VLS included female gender (*P* < 0.01; OR = 2.08), and having a fixed home address (*P* = 0.014; OR = 1.76), defined as no change in physical residence within the 12 months prior to data collection. These two variables exhibited good suppression across all levels of VLS, except in the categories of fixed home addresses with an LLV of 13% compared to 12.9% among those with non-fixed home addresses, as demonstrated in Figure 3 and Figure 4.

On the other hand, factors such as district of residence (*P* = 0.54), type of treatment (*P* = 0.27), age (*P* = 0.9), year of initiating ART medication (*P* = 0.12), and having an updated and documented cell phone number in the ePMS (*P* = 0.37), showed no significant associations with VLS in Table 1.

## Discussion

This study sought to determine the prevalence of VLS and factors associated with the suppression among OALHIV and YALHIV aged 15 to 24 years old on ART in the seven high-burden districts in Namibia. The overall VLS, according to the WHO definition,^1^ was 84.8%, which remains below the Joint UNAIDS target of 95%.^21^ Similar results were reported by the Centers for Disease Control and Prevention in seven African countries, with a VLS of 81.5% among adolescent girls and young women aged 15 to 24 years.^22^ However, the VLS found in this study surpassed the outcome of the study conducted in Rwanda on the suppression and associated factors among YALHIV at Muhima District Hospital at 71%.^23^ This might be attributed to the peer support intervention implemented in high-burden regions of Namibia, which offers psychosocial support and adherence counselling at both community and facility levels. The ministry should strengthen and expand peer support interventions and psychosocial adherence programmes to the remaining health facilities, as these have shown a positive impact on VLS among OALHIV and YALHIV. Furthermore, 71.8% of OALHIV and YALHIV are on a dolutegravir-based regimen, which is considered a robust option for achieving rapid VLS. Increasing access to these regimens through treatment optimisation will help close the viral suppression gap and accelerate progress toward achieving the UNAIDS targets.

The study revealed a higher percentage of female OALHIV and YALHIV (63.3%) compared to male patients, highlighting the elevated prevalence of HIV among adolescent girls and young women in both Namibia and worldwide.^24^ Furthermore, the study demonstrated an association between gender and VLS,^25^ although other studies did not identify such an association,^26^ possibly attributable to the equal level of services received by these population groups. Male patients were found to be less likely to have VLS compared to female patients, which aligns with the findings of numerous other studies.^27,28^ Several studies indicate that men are less likely to achieve VLS, likely because of their poorer healthcare-seeking behaviour.^28^ Targeted interventions should be developed to address the lower VLS among male OALHIV and YALHIV, focusing on improving healthcare-seeking behaviour. Additionally, HIV prevention and treatment strategies should continue to prioritise adolescent girls and young women, given their higher prevalence of HIV.

Based on the sampling methodology, a higher proportion of YALHIV records were on ART compared to OALHIV records. This is because of the aging of perinatally infected infants who were put on treatment during their childhood,^26^ as well as those who acquired the infection through sexual means and are now seeking ART to maintain their health.^29^ Even though a greater number of YALHIV are receiving ART, OALHIV exhibit a higher rate of VLS compared to their young adult counterparts.^26^ This result may be attributed to the transition from adolescence to adulthood among the young adult population.^30^ HIV programmes should strengthen transition support for YALHIV to improve adherence and VLS during the shift from adolescence to adulthood.

During the data collection period, a higher number of OALHIV and YALHIV were already on a regimen based on dolutegravir. This particular treatment approach was introduced in mid-2019, following the WHO’s endorsement of dolutegravir as the most effective ART for adolescents experiencing adherence challenges.^31^ This study demonstrates a strong association between dolutegravir and VLS among these age groups. Similar outcomes were reported in the study on the effect of dolutegravir-based regimens on the level of adherence and VLS in resource-limited settings.^32^ Dolutegravir is an integrase inhibitor that has a high genetic barrier to developing HIV drug resistance, and few drug-drug interactions compared with other ART medications.^33^

Dolutegravir-based regimens are more effective than non-dolutegravir-based regimens, as demonstrated in this study, and supported by the findings from the ODYSSEY trial.^34,35^ These findings confirm that dolutegravir-based regimens can significantly improve VLS in adolescents and young adults.^36^ Policymakers should prioritise optimising treatment strategies based on dolutegravir, a highly effective ART medication, and implementing gender-specific strategies to address the unique challenges faced by male patients in closing the gaps in VLS.

In Namibia, the ART regimens are classified into three categories: the initial treatment, representing the first-line regimen; the switch, which is the change from the first line to the second line or the third line, which is considered as a salvage therapy; and the substitution between regimen lines.^37^ Despite a higher number of regimen substitutions and overall high VLS, no association was found between treatment regimen lines (first, second, or third) and VLS, in contrast to findings from other studies that reported such an association.^38^ The high VLS observed among substitution categories can be attributed to the selection of optimised regimens for individuals who were already fully suppressed prior to the transition. More transitions were based on a dolutegravir-based regimen which sustained the VLS.^32^

A greater proportion of OALHIV and YALHIV were identified as having a fixed home address. In conjunction with this discovery, OALHIV and YALHIV with a stable or fixed home address exhibited a higher rate of VLS compared to those without a fixed home address or residence. This finding demonstrated an association between a fixed address and VLS. No research has specifically addressed these observations; nevertheless, it is plausible that the lack of studies on this matter may be because of the potential correlation between OALHIV and YALHIV with a fixed home address, and the ongoing and strong support derived from the same household.^39^

However, some studies have demonstrated that treatment supporters’ responsibilities go beyond supporting adherence to ART, to include supporting daily needs of individuals living with HIV, including clinic visits.^40^ This social supportive relationship may lead to good adherence behaviour.^41^ Older adolescents living with HIV and YALHIV with fixed home addresses can benefit from the same support in the long run compared to OALHIV and YALHIV who change addresses with the possibility of changing the treatment supporter at the same time. Household- and community-based support systems should be strengthened to support OALHIV and YALHIV, especially those without a fixed home address, to enhance adherence and VLS. Stable living situations and consistent treatment supporters play a key role in sustaining long-term treatment outcomes.

A greater number of OALHIV and YALHIV commenced their treatment before 2010, with the second-largest group initiating treatment between 2010 and 2018, and the smallest cohort beginning their treatment from mid-2019 onwards, which was mostly based on dolutegravir. The 2019 cohort exhibited a higher rate of VLS (90.1%) compared to the first and second cohorts, where both of the later groups had an equal level of suppression (85.4%). While there is no association between the year of initiating ART and VLS (*P* = 0.16), lower suppression observed in the first and second cohorts may be linked to factors like pill fatigue,^42^ resistance occurrences,^43^ and the complexity of regimens, particularly second- and third-line treatments,^44^ which may not be as effective in suppressing the virus. Treatment optimisation through transitioning patients from older regimens to more effective options, such as dolutegravir, is essential to improve VLS. Additionally, addressing challenges such as pill fatigue and drug resistance is critical to enhancing treatment outcomes for long-term ART recipients.

OALHIV and YALHIV who owned a cellphone exhibited higher VLS compared to those without a cellphone, but this was not statistically significant. The higher suppression in those with a cellphone may be attributed to the social support received from family or guardians,^40^ and the use of cellphones as reminders.^45^ The assessment of the extent of VLS was conducted based on the available variables in the ePMS; the authors acknowledge the need for further research exploring various challenges and issues impacting the adherence of OALHIV and YALHIV. These challenges encompass psychosocial and economic barriers that hinder adherence among OALHIV and YALHIV.^46^ The integration of digital tools, such as cellphone reminders, to support adherence among OALHIV and YALHIV should be considered, while also addressing broader psychosocial and economic barriers through targeted interventions. Further research is needed to better understand and address the challenges affecting adherence in these populations.

### Strengths and limitations of the study

This cross-sectional study utilised records of OALHIV and YALHIV who are representative of the broader population. The records were not selected based on VLS status or exposure to specific risk factors. This study design is appropriate for identifying initial associations and estimating the prevalence of either exposures or outcomes, such as VLS, within a population group. The study design used in this research did not allow for the establishment of causal relationships. Additionally, limitations within the database restricted access to a broader range of variables, thereby constraining the ability to explore associations with VLS.

Variables such as adherence, socio-economic status, psychosocial factors, environmental influences, and healthcare-related factors were not assessed, as they were not captured in the ePMS. Additionally, treatment regimen lines intended to demonstrate their association with VLS were misclassified in the database, preventing their use in the analysis. Despite these limitations, the researchers were satisfied with the findings, having successfully identified associations between a limited set of available variables and VLS.

### Recommendations

The national HIV/AIDS response should prioritise expanding peer support and psychosocial adherence programmes across all health facilities, because these have proven effective in improving VLS among OALHIV and YALHIV. Additionally, treatment optimisation, especially transitioning to dolutegravir-based regimens, is essential to close the VLS gap and achieve UNAIDS targets. To further improve outcomes, targeted interventions must address lower VLS among male patients by overcoming healthcare barriers and implementing gender-specific strategies. Moreover, HIV programmes should continue prioritising adolescent girls and young women while strengthening support for YALHIV during their transition to adulthood to enhance adherence.

Furthermore, household and community support systems require reinforcement, particularly for those without stable housing, since stable living conditions and consistent treatment supporters are vital for long-term adherence. Digital tools, such as cellphone reminders, can also support adherence alongside efforts to address psychosocial and economic barriers. Challenges such as pill fatigue and drug resistance must not be overlooked. Because VLS remain below UNAIDS targets, ongoing monitoring, evaluation, and research are critical to identify barriers and tailor programmes effectively. Engaging OALHIV, YALHIV, and healthcare providers in a positive, nonjudgemental manner will yield valuable insights. Finally, future research should focus on psychosocial factors especially among male patients and unstably housed individuals, and assess the long-term impact of dolutegravir-based regimens in resource-limited settings.

## Conclusion

In this study, the VLS among OALHIV and YALHIV was relatively low, at 82.4%, as compared to the set target by UNAIDS. A higher rate of VLS was associated with female gender, being on a dolutegravir-based regimen, and having a fixed home address. The WHO and UNAIDS recommend that countries working towards ending HIV/AIDS should achieve a VLS of 90% by 2020 and 95% by 2030. These findings align with numerous other studies indicating VLS falling below the target set by UNAIDS. To address the gaps, special attention should be directed towards OALHIV and YALHIV facing challenges in suppressing their VL, as well as individuals with a history of high VL, sub-optimal adherence, and those on second- or third-line regimens. Policymakers should prioritise the implementation of treatment optimisation based on dolutegravir and strategies to enhance VLS among these age groups in Namibia to align with the 95–95–95 UNAIDS targets.

## References

[1] World Health Organization (WHO). The role of HIV viral suppression in improving individual health and reducing transmission [homepage on the internet]. 2023 [cited 2024 Apr 22]. Available from: https://www.who.int/publications/i/item/9789240055179

[2] Gordon TP, Talbert M, Mugisha MK, Herbert AE. Factors associated with HIV viral suppression among adolescents in Kabale district, South Western Uganda. PLoS One. 2022;17(8):1–20. 10.1371/journal.pone.0270855 PMC9387807 35980902

[3] Winpenny EM, Winkler MR, Stochl J, van Sluijs EMF, Larson N, Neumark-Sztainer D. Associations of early adulthood life transitions with changes in fast food intake: A latent trajectory analysis. Int J Behav Nutr Phys Act. 2020;17(1):130. 10.1186/s12966-020-01024-4 33036629 PMC7547405

[4] Jiamsakul A, Kariminia A, Althoff KN, et al. HIV viral load suppression in adults and children receiving antiretroviral therapy – Results from the IeDEA collaboration. Acquir Immune Defic Syndr. 2017;76(3):319–329. 10.1097/QAI.0000000000001499 PMC5634924 28708808

[5] Hanners EK, Benitez-Burke J, Badowski ME. HIV: How to manage low-level viraemia in people living with HIV. Drugs Context. 2022;11:2021-8-13. 10.7573/dic.2021-8-13 PMC8903876 35310296

[6] Romo ML, Edwards JK, Semeere AS, et al. Viral load status before switching to dolutegravir-containing antiretroviral therapy and associations with human immunodeficiency virus treatment outcomes in sub-Saharan Africa. Clin Infect Dis. 2022;75:630–637. 10.1093/cid/ciab1006 34893813 PMC9464076

[7] Djiyou ABD, Penda CI, Madec Y, et al. Viral load suppression in HIV-infected adolescents in Cameroon: Towards achieving the UNAIDS 95% viral suppression target. BMC Pediatr. 2023;23(1):119. 10.1186/s12887-023-03943-0 36922769 PMC10015512

[8] Munyayi FK, Van Wyk B. Closing the HIV treatment gap for adolescents in Windhoek, Namibia: A retrospective analysis of predictors of viral non-suppression. Int J Environ Res Public Health. 2020;19(22):14710. 10.3390/ijerph192214710 PMC9690371 36429431

[9] Joint United Nations Programme on HIV/AIDS UNAIDS. Global HIV & AIDS statistics – Fact sheet [homepage on the internet]. 2021 [cited 2024 Jan 22]. Available from: https://www.unaids.org/en/resources/fact-sheet

[10] United Nations Children’s Fund UNICEF. HIV statistics – Global and regional trends [homepage on the internet]. 2021 [cited 2024 Jan 19]. Available from: https://data.unicef.org/topic/hivaids/global-regional-trends.

[11] Joint United Nations Programme on HIV/AIDS (UNAIDS). Namibia HIV sub-national estimates viewer. 2025 [cited 2025 Aug 17]. Available from: https://naomi-spectrum.unaids.org/

[12] President’s Emergency Plan for AIDS Relief PEPFAR. Namibia Country Operational Plan 2022 strategic direction summary [homepage on the internet]. 2022 [cited 2024 Apr 22]. Available from: https://www.state.gov/wp-content/uploads/2022/09/Namibia-COP22-SDS.pdf

[13] Ministry of Health and Social Services Directorate of Special Programmes. National strategic framework for HIV and AIDS response in Namibia 2017/18 to 2021/22 [homepage on the internet]. 2021 [cited 2024 Mar 25]. Available from https://www.unaids.org/sites/default/files/country/documents/NAM_2018_countryreport.pdf

[14] United States President’s Emergency Plan for AIDS Relief PEPFAR. Namibia country operational plan (COP) 2020 strategic direction summary [homepage on the Internet]. 2020 [cited 2024 Mar 13]. Available from: https://www.state.gov/wp-content/uploads/2020/07/COP-2020-Namibia-SDS-FINAL.pdf

[15] Center for Disease and Control CDC. CDC-Namibia [homepage on the Internet]. 2021 [cited 2024 Jan 22]. Available from: https://www.cdc.gov/globalhealth/countries/namibia

[16] Ministry of Health and Social Services. ‘Quarterly Bulletin April to June 2021 (Quarter 1)’, Response monitoring and evaluation subdivision. Windhoek: Directorate of Special Programmes; 2021.

[17] Zangirolami-Raimundo J, Echeimberg JO, Leone C. Research methodology topics: Cross-sectional studies. J Hum Growth Dev. 2018;28(3):356–360. 10.7322/jhgd.152198

[18] Majid U. Research fundamentals: Study design, population, and sample size. URNCST. 2018;2(1):1–12. 10.26685/urncst.16

[19] Amrhein V, Trafimow D, Greenland S. Inferential statistics as descriptive statistics: There is no replication crisis if we don’t expect replication. Am Stat. 2019;73(1):262–270. 10.1080/00031305.2018.1543137

[20] Ridzuan F, Wan Zainon WMN. A review of data cleansing methods for big data. Procedia Comput Sci. 2019; 161:731–738. 10.1016/j.procs.2019.11.177

[21] Joint United Nations Programme on HIV/AIDS UNAIDS. 2021. Fast-Track – Ending the AIDS epidemic by 2030 [homepage on the Internet]. 2021 [cited 2024 Jan 19]. Available from: https://www.unaids.org/en/resources/documents/2014/JC2686_WAD2014report

[22] Brown K, Williams DB, Kinchen S, et al. Status of HIV epidemic control among adolescent girls and young women aged 15–24 years – Seven African countries, 2015–2017. MMWR Morb Mortal Wkly Rep. 2018;67(1):29–32. 10.15585/mmwr.mm6701a6 29329280 PMC5769792

[23] Nyirabatsinda E, Nsanzabera C, Mochama M, Kubahoniyesu T. Viral load suppression and associated factors among young adults living with HIV at Muhima District Hospital. Cognizance J Multidiscip Stud. 2023;3(9):9–25. 10.47760/cognizance.2023.v03i09.002

[24] Joint United Nations Programme on HIV/AIDS (UNAIDS). HIV prevention among adolescent girls and young women: Fast-tracking HIV prevention among adolescent girls and young women and including boys & men [homepage on the Internet]. 2016 [cited 2024 Mar 23]. Available from: https://www.unaids.org/sites/default/files/media_asset/UNAIDS_HIV_prevention_among_adolescent_girls_and_young_women.pdf

[25] Palmer A, Gabler K, Rachlis B, et al. Viral suppression and viral rebound among young adults living with HIV in Canada. Medicine. 2018;97(22):1–10. 10.1097/md.0000000000010562 PMC6392935 29851775

[26] Mabizela S, Van Wyk B. Viral suppression among adolescents on HIV treatment in the Sedibeng District, Gauteng province. Curationis. 2022;45(1):1–8. 10.4102/curationis.v45i1.2312 PMC9575385 36226955

[27] Brown WE, Malagala H, Bajunirwe F. Social support, gender and pill burden influence viral load suppression among HIV-infected adolescents and young adults in rural south-western Uganda. Vulnerable Child Youth Stud. 2020;6:86. 10.1080/17450128.2020.1842954 PMC11563049 39544698

[28] Desta AA, Woldearegay TW, Futwi N, et al. HIV virological non-suppression and factors associated with non-suppression among adolescents and adults on antiretroviral therapy in northern Ethiopia: A retrospective study. BMC Infect Dis. 2020;20:4. 10.1186/s12879-019-4732-6 31898535 PMC6941313

[29] Maskew M, Bor J, MacLeod W, Carmona S, Sherman GG. The adolescent HIV treatment bulge in South Africa’s National HIV Program: A retrospective cohort. Lancet HIV. 2019;6(11):760–768. 10.1016/S2352-3018(19)30234-6 PMC7119220 31585836

[30] Ashaba S, Zanoni BC, Baguma C, et al. Challenges and fears of adolescents and young adults living with HIV facing transition to adult HIV care. AIDS Behav. 2023;27:1189–1198. 10.1007/s10461-022-03856-6 36129557 PMC10027623

[31] Bacha JM, Dlamini S, Anabwani F, et al. Realizing the promise of dolutegravir in effectively treating children and adolescents living with HIV in real-world settings in 6 countries in Eastern and Southern Africa. Pediatr Infect Dis. 2023;42(7):576–581. 10.1097/INF.0000000000003878 PMC10259212 36795586

[32] Ezenwosu IL, Onu JU, Chukwuma UV, et al. Effect of dolutegravir-based drug combinations on the level of medication adherence and viral load among adolescents living with HIV in a resource-limited setting: A pre-post design. Int J Adolesc Med Health. 2023;35(6):457–465. 10.1515/ijamh-2023-0082 38059505

[33] Cottrell ML, Hadzic T, Kashuba AD. Clinical pharmacokinetic, pharmacodynamic and drug-interaction profile of the integrase inhibitor dolutegravir. Clin Pharmacokinet. 2013;52(11):981–994. 10.1007/s40262-013-0093-2 23824675 PMC3805712

[34] Moore CL, Turkova A, Mujuru H, et al. ODYSSEY clinical trial design: A randomised global study to evaluate the efficacy and safety of dolutegravir-based antiretroviral therapy in HIV-positive children, with nested pharmacokinetic sub-studies to evaluate pragmatic WHO-weight-band based dolutegravir dosing. BMC Infect Dis. 2021;21:5. 10.1186/s12879-020-05672-6 33446115 PMC7809782

[35] Yan L, Henegar CE, Marconi VC, et al. Effectiveness of dolutegravir-based regimens compared to raltegravir-, elvitegravir-, bictegravir-, and darunavir-based regimens among older adults with HIV in the Veterans Aging Cohort Study (VACS). AIDS Res Ther. 2024;21:96. 10.1186/s12981-024-00681-w 39709467 PMC11662819

[36] Mutagonda RF, Mlyuka HJ, Maganda BA, Kamuhabwa AAR. Adherence, effectiveness and safety of dolutegravir based antiretroviral regimens among HIV infected children and adolescents in Tanzania. J Int Assoc Provid AIDS Care. 2022;21:23259582221109613. 10.1177/23259582221109613 35776522 PMC9257168

[37] Wood BR, Spach DH. Switching or simplifying antiretroviral therapy. National HIV Curriculum [homepage on the Internet]. 2023 [cited 2024 Apr 12]. Available from: https://www.hiv.uw.edu/go/antiretroviral-therapy/switching-antiretroviral-therapy/core-concept/all

[38] Fekadu G, Bati L, Gebeyehu H. Reasons for antiretroviral treatment change among adult HIV/AIDS patients at Nedjo General Hospital, Western Ethiopia. Open AIDS J. 2019;13:65–73. 10.2174/1874613601913010065

[39] Onyango B, Mokaya R, Wasianga J, et al. Factors associated with viral load suppression among orphans and vulnerable children and adolescents living with HIV in Kenya. PLOS Glob Public Health. 2023;3(3):1–13. 10.1371/journal.pgph.0000794 PMC10035747 36963026

[40] Bezabhe WM, Chalmers L, Bereznicki LR, Peterson GM, Bimirew MA., Kassie DM. Barriers and facilitators of adherence to antiretroviral drug therapy and retention in care among adult HIV-positive patients: A qualitative study from Ethiopia. PLoS One. 2014;9(5):97353. 10.1371/journal.pone.0097353 PMC4020856 24828585

[41] Knowlton AR, Yang C, Bohnert A, Wissow L, Chander G., Arnsten JA. Informal care and reciprocity of support are associated with HAART adherence among men in Baltimore, MD, USA. AIDS Behav. 2011;15(7):1429–1436. 10.1007/s10461-010-9749-1 20632081 PMC3006496

[42] Kawuma R, Bernays S, Siu G, Rhodes T, Seeley J. ‘Children will always be children’: Exploring perceptions and experiences of HIV-positive children who may not take their treatment and why they may not tell. Afr J AIDS Res. 2014;13(2):189–195. 10.2989/16085906.2014.927778 25174636

[43] Miti S, Handema R, Mulenga L, et al. Prevalence and characteristics of HIV drug resistance among antiretroviral treatment (ART) experienced adolescents and young adults living with HIV in Ndola, Zambia. PLoS One. 2020;15(8):2. 10.1371/journal.pone.0236156 PMC7430722 32804970

[44] Mwangi A, Van Wyk B. Factors associated with viral suppression among adolescents on antiretroviral therapy in Homa Bay County, Kenya: A retrospective cross-sectional study. HIV/AIDS – Res Palliat Care. 2021;13:1111–1118. 10.2147/hiv.s345731 PMC8713714 34992469

[45] Kamangu, J. HIV programme to improve adherence among older adolescents and younger adults living with HIV in Namibia [unpublished dissertation]. College of Human Science, Pretoria: University of South Africa; 2024.

[46] Adraro W, Abeshu G, Abamecha F. Physical and psychological impact of HIV/AIDS toward youths in Southwest Ethiopia: A phenomenological study. BMC Public Health. 2024;24:2963. 10.1186/s12889-024-20478-w 39456003 PMC11506255

